# Placental mitochondrial DNA and *CYP1A1* gene methylation as molecular signatures for tobacco smoke exposure in pregnant women and the relevance for birth weight

**DOI:** 10.1186/s12967-016-1113-4

**Published:** 2017-01-04

**Authors:** Bram G. Janssen, Wilfried Gyselaers, Hyang-Min Byun, Harry A. Roels, Ann Cuypers, Andrea A. Baccarelli, Tim S. Nawrot

**Affiliations:** 1 Centre for Environmental Sciences, Hasselt University, Hasselt, Belgium; 2 Department of Obstetrics, East-Limburg Hospital, Genk, Belgium; 3 Department of Physiology, Hasselt University, Diepenbeek, Belgium; 4 Laboratory of Environmental Epigenetics, Exposure Epidemiology and Risk Program, Harvard School of Public Health, Boston, MA 02215 USA; 5 Louvain Centre for Toxicology and Applied Pharmacology (LTAP), Université Catholique de Louvain, Brussels, Belgium; 6 Department of Environmental Health Sciences, Mailman School of Public Health, Columbia University, New York, NY 10032 USA; 7 Department of Public Health & Primary Care, Occupational and Environmental Medicine, Leuven University, Louvain, Belgium; 8 Centre for Environmental Sciences, Hasselt University, Agoralaan Gebouw D, 3590 Diepenbeek, Belgium

**Keywords:** Birth weight, *CYP1A1*, Epigenetics, DNA methylation, Mitochondrial DNA content, Mitochondrial DNA methylation, Placental tissue, Tobacco smoke

## Abstract

**Background:**

Maternal smoking during pregnancy results in an increased risk of low birth weight through perturbations in the *utero*-placental exchange. Epigenetics and mitochondrial function in fetal tissues might be molecular signatures responsive to in utero tobacco smoke exposure.

**Methods:**

In the framework of the ENVIR*ON*AGE birth cohort, we investigated the effect of self-reported tobacco smoke exposure during pregnancy on birth weight and the relation with placental tissue markers such as, (1) relative mitochondrial DNA (mtDNA) content as determined by real-time quantitative PCR, (2) DNA methylation of specific loci ﻿of mtDNA (*D-loop* and *MT-RNR1*), and (3) DNA methylation of the biotransformation gene *CYP1A1* (the last two determined by bisulfite-pyrosequencing). The total pregnant mother sample included 255 non-smokers, 65 former-smokers who had quit smoking before pregnancy, and 62 smokers who continued smoking during pregnancy.

**Results:**

Smokers delivered newborns with a birth weight on average 208 g lower [95% confidence interval (CI) −318 to −99, *p* = 0.0002] than mothers who did not smoke during pregnancy. In the smoker group, the relative mtDNA content was lower (−21.6%, 95% CI −35.4 to −4.9%, *p* = 0.01) than in the non-smoker group; whereas, absolute mtDNA methylation levels of *MT-RNR1* were higher (+0.62%, 95% CI 0.21 to 1.02%, *p* = 0.003). Lower CpG-specific methylation of *CYP1A1* in placental tissue (−4.57%, 95% CI −7.15 to −1.98%, *p* < 0.0001) were observed in smokers compared with non-smokers. Nevertheless, no mediation of *CYP1A1* methylation nor any other investigated molecular signature was observed for the association between tobacco smoke exposure and birth weight.

**Conclusions:**

mtDNA content, methylation of speci﻿fic loci of mtDNA, and *CYP1A1* methylation in placental tissue may serve as molecular signatures for the association between gestational tobacco smoke exposure and low birth weight.

**Electronic supplementary material:**

The online version of this article (doi:10.1186/s12967-016-1113-4) contains supplementary material, which is available to authorized users.

## Background

A growing area of research interest with major public health implications are the consequence of insults during fetal life for the health status in child- and adulthood. It is well known that maternal smoking during pregnancy increases the risk of low birth weight [1, 2] and preterm delivery [3, 4] which is probably due to perturbations in the fetoplacental exchange [5]. The exact mechanism(s) underlying these adverse effects remain unclear, but emerging data suggests that biochemical, genetic, and epigenetic processes respond to and/or are modified by in utero tobacco exposure of the fetal organism.

Tobacco smoke consists of particulate and gaseous phases containing more than 7000 chemicals of which at least 70 substances are known to cause cancer [6]. Constituents of tobacco smoke such as polycyclic aromatic hydrocarbons (PAHs) enter cells and may activate genes involved in detoxification processes such as *CYP1A1* (cytochrome P450, family 1, subfamily A, polypeptide 1) via the aryl hyrdrocarbon receptor (Ahr) signaling pathway resulting in an oxidative imbalance of the cells. Mitochondrial DNA (mtDNA), which resides as multiple double stranded circular copies in mitochondria, is extremely vulnerable and responsive to tobacco-induced oxidative stress [7–9]. As a result, alterations in mtDNA content, characterized as increasing or decreasing mtDNA copies, are an indication of dysfunctional or damaged mitochondria [10]. The inter-genomic crosstalk between mitochondria and the nucleus is complex. Growing evidence suggests that mitochondrial dysfunction may affect the epigenetic landscape of the nuclear genome [11, 12]. DNA methylation is the most intensively studied epigenetic modification. Exposures to adverse environmental factors are important determinants for methylation programming during early life [13, 14]. Global [15–18] and gene-specific (e.g. *CYP1A1*) [19–26] DNA methylation differences have been demonstrated in cord blood and placental cells of neonates from mothers who smoked during pregnancy. Disruption of the fetal methylome has been associated with adverse pregnancy outcomes and could provide an underlying mechanism through which smoking affects fetal growth [20, 24, 27].

While several studies described separately the effect of maternal smoking during pregnancy on birth weight, mitochondrial DNA, and *CYP1A1* methylation, we integrated these biological endpoints in our investigation of placental tissue collected in the framework of the ENVIR*ON*AGE birth cohort study [28]. We hypothesized that exposure to tobacco smoke during pregnancy impacts birth weight and concomitantly also these molecular signatures.

## Methods

### Study population

In the present study, 382 mother-newborn pairs were enrolled in the ENVIR*ON*AGE birth cohort in Belgium (acronym for ENVIRonmental influence *ON* AGEing in early life). All procedures were approved by the Ethical Committee of Hasselt University and East-Limburg Hospital. The study design and procedures were previously described in detail [29]. Briefly, written informed consent was obtained from each participating mother who gave birth in the East-Limburg Hospital in Genk, Belgium. For this study, the only inclusion criterion was that mothers had to be able to fill out questionnaires in Dutch. Enrolment was equally spread over all seasons of the year. Questionnaires and medical records were consulted after birth and provided information on maternal age, maternal education, smoking status, ethnicity, pre-pregnancy body mass index (BMI), gestational age, newborn’s sex, Apgar scores, birth weight and length, parity, and ultrasonographic data. Maternal education was coded as “low” (no diploma or primary school), “middle” (high school) or “high” (college or university degree). Based on the native country of the newborn’s grandparents we classified his/her ethnicity as European-Caucasian when two or more grandparents were European, or non-European when at least three grandparents were of non-European origin. We asked the mothers whether they consumed alcohol during pregnancy, used medication, and how many times per week they practiced physical exercises for at least 20 min. Information about tobacco smoke exposure was collected by self-report of the mothers. They were asked whether they continued smoking during pregnancy (smoker group, *n* = 62), whether they smoked before pregnancy and stopped when pregnant (past-smoker group, *n* = 65), or whether they never smoked in their life (non-smoker group, *n* = 255). Mothers who had ever smoked filled out the number of smoking years and the number of cigarettes smoked per day before and during pregnancy. We also asked the mothers how long (months) they continued smoking before becoming aware of being pregnant. Furthermore, we have data on passive smoke exposure (due to indoor smoking by somebody else).

### Sample collection

Placentas were deep-frozen within 10 min after delivery. Specimens of placental tissue were taken on minimally thawed placentas for DNA extraction. We took villous tissue (1–2 cm^3^) at a fixed location from the fetal side of the placenta, approximately 1–1.5 cm below the chorio-amniotic membrane, and preserved the biopsies at −80 °C [30]. At a later stage, genomic DNA was isolated from the placental biopsies using the QIAamp DNA mini kit (Qiagen, Inc., Venlo, Netherlands) and stored at −80 °C until further use.

### DNA methylation analysis

We performed DNA methylation analysis by highly quantitative bisulfite polymerase chain reaction (PCR) pyrosequencing as previously described in detail [30]. Bisulfite conversions were performed using 1 µg of extracted genomic DNA with the EZ-96 DNA methylation Gold kit (Zymo Research, Orange, CA, USA) according to the manufacturer’s instructions. We examined four CpG sites within the promoter region of the *CYP1A1* gene and for the mitochondrial genome we examined two CpG sites in the *MT*-*RNR1* region, and three CpG sites in the *D*-*loop* region. Detailed information regarding primer sequences is given in Additional file 1: Table S1. Prior to pyrosequencing, PCR amplification of regions of interest was performed in a total reaction volume of 30 µl, containing 15 µl GoTaq Hot Start Green Master Mix (Promega, Madison, WI, USA), 10 pmol forward primer, 10 pmol reverse primer, 1 µl bisulfite-treated genomic DNA, and water. PCR products were purified and sequenced by pyrosequencing using the PyroMark Q96 MD Pyrosequencing System (Qiagen, Inc., Germantown, MD, USA). The degree of methylation was expressed as the ratio (percentage) of methylated cytosines over the sum of methylated and unmethylated cytosines. The efficiency of the bisulfite-conversion process was assessed using non-CpG cytosine residues within the sequence. We used 0% (PSQ-T oligo: 5′-TTGCGATACAACGGGAACAAACGTTGAATTC-3′) and 100% (PSQ-C oligo: 5′-TTGCGATACGACGGGAACAAACGTTGAATTC-3′) DNA methylation control oligos. The sequencing primer for the control oligo was: 5′-AACGTTTGTTCCCGT-3′. We mixed the PSQ-C oligo (or PSQ-T oligo) with the sequencing oligo in PyroMark Annealing Buffer (Qiagen, Inc., Valencia, CA, USA) and performed pyrosequencing with the sequencing entry C/TGTAT. We assessed the within-placenta variability in a random subset of 19 placentas as previously described [30]. The between-placenta variability was higher than the within-placenta variability for *CYP1A1* (58 vs. 42%, *p* < 0.0001), the *D*-*loop* region (61 vs. 39%, *p* = 0.01), and *MT*-*RNR1* (58 vs. 42%, *p* = 0.009).

### Mitochondrial DNA content analysis

The mtDNA content was measured by determining the ratio of two mitochondrial gene copy numbers (*MTF3212/R3319* and *MT*-*ND1*) to two single-copy nuclear control genes (*RPLP0* and *ACTB*) using a quantitative real-time PCR (qPCR) assay as previously described [29] and used with a small modification. Isolated genomic DNA (12.5 ng) was added to 7.5 µl mastermix consisting of Fast SYBR^®^ Green I dye 2x (5 µl/reaction), forward and reverse primer (each 0.3 µl/reaction), and RNase free water (1.9 µl/reaction) for a final volume of 10 µl per well. Primer sequences (Additional file 1: Table S1) were diluted to a final concentration of 300 nM in the master mix. Samples were run in triplicate in a 384-well format. Real-time PCR was performed using the 7900HT Fast Real-Time PCR System (Applied Biosystems, Foster City, CA, USA) with the following thermal cycling profile: 20 s at 95 °C (activation), followed by 40 cycles of 1 s at 95 °C (denaturation) and 20 s at 60 °C (annealing/extension), and ending with melting curve analysis (15 s at 95 °C, 15 s at 60 °C, 15 s at 95 °C). qBase software (Biogazelle, Zwijnaarde, Belgium) was used to normalize data and correct for run-to-run differences [31].

### Statistical analysis

We used SAS software (version 9.2; SAS Institute Inc., Cary, NC, USA) for database management and statistical analysis. Relative mtDNA content (unitless) was log_10_-transformed to normalize the distribution. The relationships between smoking and continuous variables were examined with one-way ANOVA procedures and Chi square tests for the categorical variables. We applied conventional multiple linear regression to estimate the association between maternal smoking status and birth weight, length, or placental mtDNA content. The pyrosequencing-based DNA methylation analysis produced a methylation value (%) for each CpG site of *CYP1A1* (four CpGs), *MT*-*RNR1* (two CpGs) and the *D*-*loop* region (three CpGs). Correlations between adjacent CpG sites within one gene or region were tested with Pearson correlation coefficients. With mixed-effects models, we took into account each CpG dinucleotide position and tested the association between gene-specific DNA methylation and maternal smoking status. We applied Dunnett’s test for multiple comparisons of smokers and past-smokers with the reference group (non-smokers). Maternal alcohol consumption, medication use, physical activity, maternal education, ethnicity, maternal age, pre-pregnancy BMI, parity, gestational age, and newborn’s sex were considered as possible confounders, but only those associated with maternal smoking (*p* ≤ 0.05) and which potentially could influence birth weight and length, mtDNA content or DNA methylation were considered for entry in the models. However, newborn’s sex, maternal age, gestational age, ethnicity, parity, and pre-pregnancy BMI were forced into the model regardless of the *p* value, in addition to maternal education, and alcohol consumption. Q–Q plots of the residuals were used to test the linearity assumption of the models.

In a sensitivity analysis, Pearson correlation coefficients were calculated between birth weight or length and measures of smoking (years of smoking, pack-year or number of cigarettes smoked per day during pregnancy). Furthermore, we used mediation analysis to investigate whether the examined molecular signatures underlie the association between gestational tobacco smoke exposure and birth weight [32].

## Results

### Participant’s demographics and lifestyle factors

Demographic characteristics and perinatal factors of 382 mother-newborn pairs are reported in Table 1. The newborns, among them 194 girls (50.8%), had a mean gestational age of 39.2 weeks (range 35–42) and comprised 200 (52.3%) primiparous and 142 (37.2%) secundiparous newborns. The mean (SD) birth weight of the newborns was 3429 (426) g and birth length 50.3 (1.9) cm. About 90% (*n* = 332) of the newborns were Europeans of Caucasian ethnicity. Mean maternal age was 29.0 years (range 18–42 years). Most women (66.7%, *n* = 255) never smoked cigarettes and 65 women (17.0%) stopped smoking before pregnancy; whereas, 62 mothers (16.2%) reported to have smoked during pregnancy [on average 7.8 cigarettes per day (inter quartile range, IQR: 5–10]. A fair number of mothers (*n* = 73, 19.1%) occasionally consumed alcohol during their pregnancy.

**Table 1:** Characteristics of mother-newborn pairs according to self-reported tobacco smoke exposure during pregnancy

Variable	All (*n* = 382)	Non-smokers (*n* = 255)	Past-smokers (*n* = 65)	Smokers (*n* = 62)	*p* value*
Newborn					
Sex					0.47
Male	188 (49.2%)	120 (47.1%)	34 (52.3%)	34 (54.8%)	
Female	194 (50.8%)	135 (52.9%)	31 (47.7%)	28 (45.2%)	
Ethnicity					0.73
European-Caucasian	332 (86.9%)	223 (87.4%)	57 (87.7%)	52 (83.9%)	
Non-European	50 (13.1%)	32 (12.6%)	8 (12.3%)	10 (16.1%)	
Gestational age, w	39.2 ± 1.2	39.3 ± 1.2	39.2 ± 1.3	39.2 ± 1.2	0.82
Birth weight, g	3429 ± 426	3472 ± 424	3437 ± 423	3247 ± 395	0.0009
Birth length, cm	50.3 ± 1.9	50.5 ± 2.0	50.5 ± 1.7	49.5 ± 1.8	0.0007
Mother					
Age, year	29.0 ± 4.7	29.4 ± 4.5	28.9 ± 4.9	27.7 ± 4.8	0.03
Pre-pregnancy BMI, kg/m^2^	24.3 ± 4.5	24.2 ± 4.4	24.8 ± 5.3	24.2 ± 4.1	0.65
Maternal education					<0.0001
Low	51 (13.3%)	26 (10.2%)	6 (9.2%)	19 (30.6%)	
Middle	131 (34.3%)	77 (30.2%)	25 (38.5%)	29 (46.8%)	
High	200 (52.4%)	152 (59.6%)	34 (52.3%)	14 (22.6%)	
Medication use^a^					0.57
None	134 (37.8%)	92 (39.5%)	21 (32.3%)	21 (37.5%)	
Alcohol consumption					0.03
Occasionally	73 (19.1%)	42 (16.5%)	20 (30.8%)	11 (17.7%)	
Physical activity (>20 min)^b^					0.40
<1 times per week	122 (33.3%)	82 (33.3%)	19 (29.7%)	21 (36.8%)	
1 times per week	86 (23.4%)	63 (25.6%)	15 (23.4%)	8 (14.0%)	
>2 times per week	159 (43.3%)	101 (41.1%)	30 (46.9%)	28 (49.2%)	
Parity					0.42
1	200 (52.3%)	132 (51.8%)	36 (55.4%)	32 (51.6%)	
2	142 (37.2%)	91 (35.7%)	25 (38.5%)	26 (41.9%)	
≥3	40 (10.5%)	32 (12.5%)	4 (6.1%)	4 (6.5%)	
Cigarettes before pregnancy	–	–	11.0 ± 6.9	10.3 ± 6.7	
Cigarettes during pregnancy	–	–	–	7.8 ± 4.6	

Data are presented as arithmetic mean ± standard deviation (SD) or number (%)

* *p* value derived from one-way ANOVA or Chi square tests in case of continuous or categorical variables respectively

^a^Medication use: occasional use of paracetamol or antibiotics (28 missing data)

^b^Missing data for 15 subjects

Compared to the non-smokers, the group of smoking mothers were younger (27.7 ± 4.8 vs. 29.0 years ± 4.7, *p* = 0.008), comprised less women with higher education (22.6 vs. 59.6%, *p* < 0.0001), and delivered newborns of lower birth weight and length. Alcohol consumption was higher in the past-smoker group than in the non-smoker group (30.8 vs. 16.5%, *p* = 0.01).

### Smoking status and birth parameters

Birth weight and length were respectively 225 g and 1 cm lower for newborns from the smoker mothers compared to the non-smokers (Table 1). After adjustment for maternal age, gestational age, newborn’s sex, maternal education, ethnicity, parity, pre-pregnancy BMI, and alcohol consumption, we still observed a lower birth weight (−208 g, 95% CI −318 to −99 g, *p* = 0.0002) and a shorter birth length (−1.0 cm, 95% CI −1.5 to −0.5 cm, *p* < 0.0001) in newborns delivered by women who continued smoking during pregnancy compared to non-smoking mothers. Mothers who stopped smoking before pregnancy delivered newborns whose birth weight (*p* = 0.55) or length (*p* = 0.87) did not differ from that of never-smokers.

### Smoking status and mtDNA in placental tissue

**Fig. 1:**
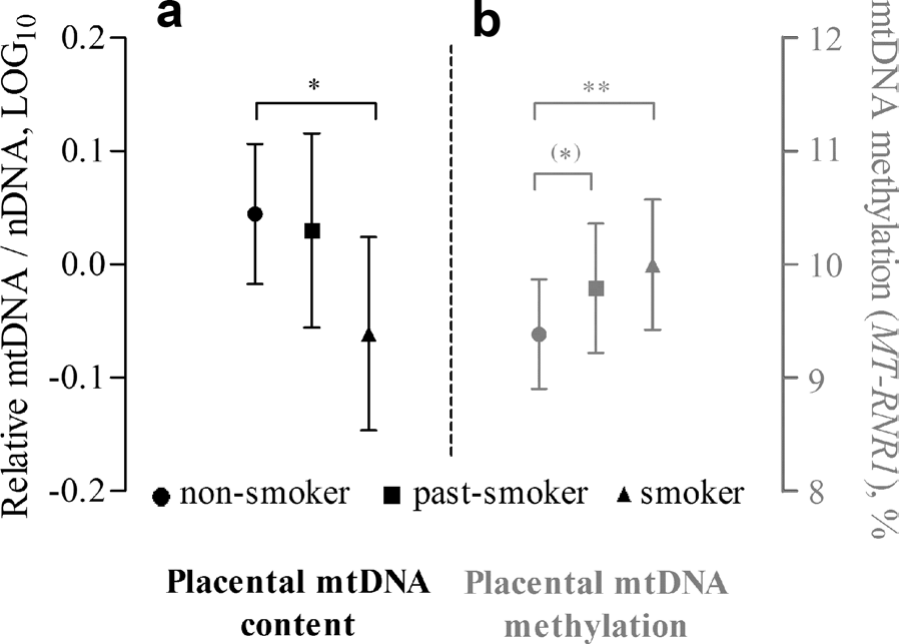
Estimated mean levels of mtDNA content and mtDNA methylation in placental tissue of non-smokers (*n* = 255), past smokers (*n* = 65), and current smokers (*n* = 62). The *bars* represent the estimated means with 95% confidence intervals for the non-smoking (*filled circle*), past-smoking (*filled square*), and smoking group (*filled triangle*). **a** Relative mtDNA content levels (unitless) are log_10_-transformed; **b** Methylation of the *MT*-*RNR1* gene are absolute methylation levels. Both the generalized linear model for mtDNA content and the mixed-effects model for mtDNA methylation were adjusted for maternal age, gestational age, newborn’s sex, maternal education, ethnicity, parity, pre-pregnancy BMI, and alcohol consumption. (*)*p* = 0.06; *p < 0.05; **p < 0.005: difference compared to the non-smoking group

After adjustment for the aforementioned covariates, the relative mtDNA content in placental tissue was 21.6% (95% CI −35.4 to −4.9, *p* = 0.01) lower in smoking mothers, but not in past-smokers (*p* = 0.72), in comparison with non-smokers (Fig. 1). In contrast, absolute methylation levels of the mitochondrial genome at the *MT*-*RNR1* gene were higher in mothers who continued smoking during pregnancy (+0.62%, 95% CI 0.21 to 1.02, *p* = 0.003) and marginally higher in mothers who stopped smoking prior to pregnancy (+0.37%, 95% CI −0.02 to 0.75, *p* = 0.06) compared with non-smokers (Fig. 1). We found no interaction between smoking status and CpG site of *MT*-*RNR1* (*p*_int_ = 0.94), and the methylation levels at the *D*-*loop* region did not differ between the groups (*p* = 0.85).

### Smoking status and gene-specific *CYP1A1* methylation in placental tissue

**Fig. 2:**
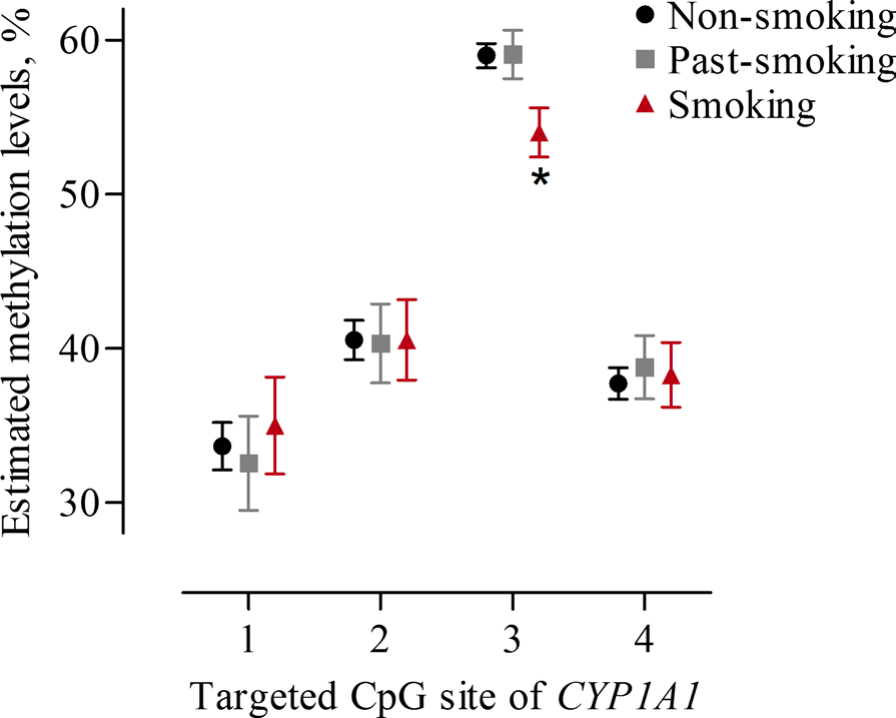
Unadjusted estimates of methylation levels in percentage (%) at four targeted CpG sites within the *CYP1A1* promoter region of placental tissue. Estimated methylation levels at each CpG are indicated for each smoking category [*black* non-smokers (*n* = 255); *grey* past-smokers (*n* = 65); *red* smokers (*n* = 62)]. The *error bars* display the 95% confidence intervals

The examined CpGs in the promoter region of *CYP1A1* showed strong inter-correlations for placental tissue (r = 0.71–0.93, *p* < 0.0001) (Additional file 1: Figure S1). Unadjusted mixed-effects models revealed an interaction effect between smoking status and CpG sites of the promoter region of *CYP1A1* (*p*_int_ < 0.0001). Placental methylation levels at CpG3 were significantly lower in mothers who continued smoking during pregnancy compared to non-smoking mothers (Fig. 2), even after adjustment for maternal age, gestational age, newborn’s sex, maternal education, ethnicity, parity, pre-pregnancy BMI, and alcohol consumption (−4.57%, 95% CI −7.15 to −1.98, *p* < 0.0001) (Table 2). No significant differences in CpG methylation levels were observed in mothers who stopped smoking before pregnancy.

**Table 2:** Effect of tobacco smoking status during pregnancy on CpG sites of *CYP1A1* in placental tissue (*n* = 382)

*CYP1A1* methylation^a^	Non-smoking	Past-smoking			Smoking		
		%	(95% CI)	*p* value	%	(95% CI)	*p* value
CpG 1	Ref.	−1.16	(−5.90 to 3.56)	0.99	1.75	(−3.09 to 6.60)	0.95
CpG 2	Ref.	−0.30	(−4.25 to 3.66)	0.99	0.43	(−3.63 to 4.49)	0.99
CpG 3	Ref.	0.02	(−2.47 to 2.51)	0.99	*−4.57*	*(−7.15 to −1.98)*	*<0.0001*
CpG 4	Ref.	1.00	(−2.11 to 4.11)	0.98	0.98	(−2.22 to 4.19)	0.99

Data shown in italic is significant

Mixed-effects models are adjusted for maternal age, gestational age, newborn’s sex, maternal education, ethnicity, parity, pre-pregnancy BMI, and alcohol consumption

^a^Estimated absolute percentage (%) change in methylation levels for each CpG of *CYP1A1* compared to the non-smoking group (reference). The 95% CI and *p* values are adjusted according to Dunnett’s procedure

### Sensitivity analysis

**Fig. 3:**
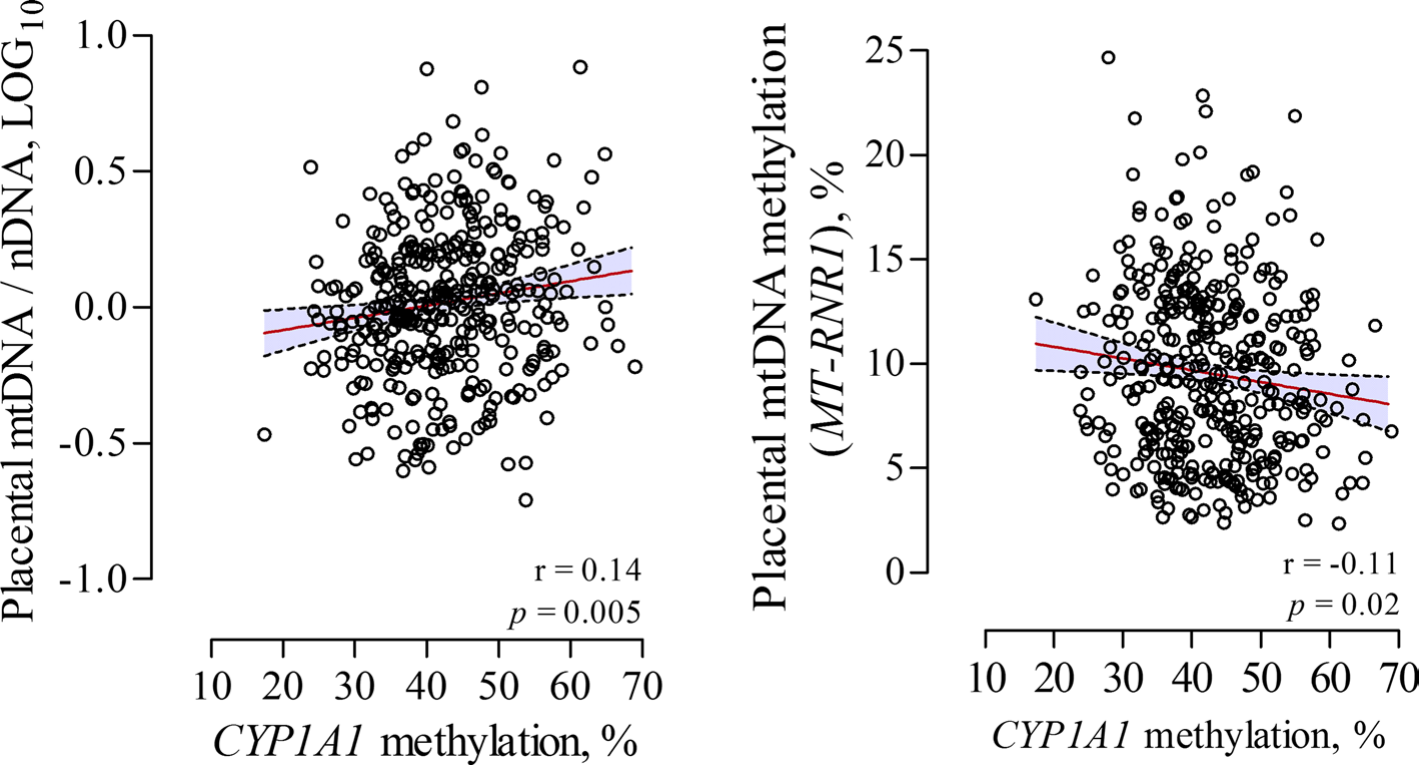
Correlation between *CYP1A1* methylation levels (%) and mtDNA content (log_10_) or mtDNA methylation (*MT-RNR1*) (%) in placental tissue. The* dashed lines* in the correlation plots depict the 95% CI

As anticipated, we observed a clear dose-effect relation between birth weight or length and measures of smoking status (years of smoking, pack-year, or the number of cigarettes smoked per day during pregnancy). In comparison with non-smokers, no significant difference was observed in birth weight or length of newborns from mothers who stopped smoking for a longer period of time before pregnancy or mothers who stopped just prior to pregnancy. We observed a positive association of *CYP1A1* methylation levels with placental mtDNA content (r = 0.14, *p* = 0.005), and a negative association with placental mtDNA methylation (r = −0.11, *p* = 0.02) (Fig. 3). Furthermore, we observed no mediation of *CYP1A1* methylation nor any other investigated molecular signature between the association of tobacco smoke exposure and birth weight (data not shown).

## Discussion

The present investigation showed that women who smoked during pregnancy had neonates with lower birth weight and length, lower mtDNA content, higher mtDNA methylation at specific loci, and lower CpG-specific methylation levels of *CYP1A1* in placental tissue.

Despite a limited number of (epi)genomic studies in placental tissue and cord blood, we are improving our understanding of the molecular pathways underlying the association between gestational tobacco smoke exposure and low birth weight. Combining gene expression and epigenome-wide methylation arrays Suter et al. [26] showed that the expression of 623 genes and the methylation of 1024 CpG dinucleotides were significantly altered in placentas of smokers. For 438 genes significant correlations were revealed between methylation and gene expression, and their potential functions or mechanisms were explored using an Ingenuity Pathway Analysis. The authors found that the gene list was enriched for genes involved in functional pathways such as mitochondrial dysfunction, oxidative phosphorylation and hypoxia. Indeed, mitochondria, the “powerhouses” of cells, provide cellular energy via oxidative phosphorylation and are very sensitive to exposures that induce oxidative stress. The double stranded circular mtDNA, of which multiple copies are present in mitochondria, is vulnerable to reactive oxygen species (ROS) because of an inefficient DNA repair capacity and close proximity to the electron transport chain [33]. The estimated mutation rate of mtDNA is 5-10 times higher compared to nuclear DNA [34]. We showed that placental mtDNA content and methylation levels were responsive to tobacco smoke exposure during pregnancy indicating that mtDNA is a sensitive marker of mitochondrial damage and dysfunction as proposed by Sahin et al. [10]. In addition to other studies reporting changes in placental mtDNA content in smoking mothers [7, 8] or mothers exposed to air pollution [29], we provide here the first epidemiological evidence of altered methylation levels at specific loci of the mitochondrial genome of placental tissue in response to tobacco smoke exposure during pregnancy. We suggest that pollution-induced epigenetic modifications of the mitochondrial genome may prime alterations in mtDNA content by regulating mitochondrial function and biogenesis [35]. Damaged or non-functioning mitochondria are specifically degraded through mitophagy and could result in a depletion of mtDNA [36], which moreover may lead to changes in methylation patterns of a number of nuclear genes [12]. The sensitivity analysis showed that mtDNA content and mtDNA methylation correlated with methylation of *CYP1A1* in placental tissue, which could be indicative of a relationship between mitochondrial dysfunction and the epigenetic landscape of the nuclear genome [11]. Whether mitochondrial dysfunction affects gene expression and methylation patterns of other genes needs to be elucidated.

**Fig. 4:**
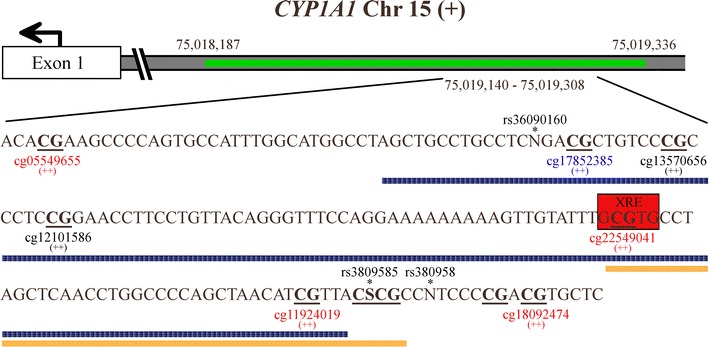
CpG sites located on the shore of a CpG island in a bidirectional regulatory region of the *CYP1A1* gene. The CpG island is depicted in* green* with a distinct portion magnified (chr15:75,019,140-75,019,308). CpG sites are denoted in* bold* and* underlined* whereas possible SNPs are indicated with an* asterisk*. The* orange bar* represents the analyzed sequence in our study and includes four CpG sites. The* blue bar* represents the analyzed sequence in placental tissue derived from the article of Suter et al. [19] and includes five CpG sites. The cg probes that were investigated in the 450 K study of Joubert et al. [23] in cord blood are displayed with the color representing the statistical significance of the association between plasma cotinine and methylation of the probe (*blue p* > 1 × 10^−5^; *black* 1 × 10^−5^ ≥ *p* ≥ 1 × 10^−7^; *red p* < 1 × 10^−7^) and the magnitude of effect (++: higher methylation). The information on the figure is based on the UCSC Genome Browser on Human Feb. 2009, GRCh37/hg19

An expanding body of evidence suggests that the epigenome of placental tissue and cord blood is sensitive to environmental exposures [13]. Epigenome-wide methylation studies are used to examine the epigenetic status of the human genome at many different *loci* in a number of individuals and also to assess whether any of these CpG *loci* are associated with a trait or an environmental pollutant [37]. A 450 K epigenome-wide methylation study by Joubert et al. [23] demonstrated differentially methylated detoxifying genes (*AHRR* and *CYP1A1*) in cord blood of newborns exposed to tobacco smoke during pregnancy. This finding was confirmed in another population of infants by analyzing whole blood obtained by a heel prick [25]. Maternal smoking as assessed by both self-report and cotinine levels in plasma showed higher methylation levels at different CpGs of *CYP1A1* in cord blood [23]. Conversely, in placental tissue of smoker mothers, Suter et al. [19] observed hypomethylated CpG dinucleotides proximal to a xenobiotic response element (XRE); whereas, those distal from such elements did not demonstrate differential methylation. The authors calculated the total percentage of methylation for a distinct region of the promoter (−1411 to −1295 bp from the transcription start site) without taking into account the separate CpGs, unlike we did in our study. We observed lower methylation levels at a specific CpG site that lies adjacent to a XRE site in placental tissue of mothers who smoked during pregnancy. It is important to note that this specific CpG site harbors a C/G single nucleotide polymorphism (SNP: rs3809585 with allele frequencies C: 1.717% and G: 98.283%). We are confident that this SNP did not affect DNA methylation since all pyrograms confirmed a G nucleotide in the analyzed sequence. Interestingly, the study of Joubert et al. [23] in cord blood, the study of Suter et al. [19] in placental tissue, and our study in placental tissue, examined approximately the same region of interest and CpGs, however with different detection methods (Fig. 4). With the bisulfite pyrosequencing approach, we confirmed hypomethylation at a specific CpG of the *CYP1A1* gene in placental tissue which is in contrast with the findings in cord blood [23]. Although we lacked meaningful gene expression data of *CYP1A1* in our study, Suter et al. [19] previously showed that lower methylation levels in a region covering the XRE site were correlated with increased expression of *CYP1A1* in placental tissue. Moreover, other studies demonstrated increased *CYP1A1* mRNA [38] and protein [39] expression in human placentas in response to tobacco smoke exposure. Constituents of tobacco smoke such as PAHs enter cells and are recognized by the aryl hydrocarbon receptor (Ahr) causing its translocation to the nucleus and the formation of a heterodimer with the Ahr nuclear translocator protein (ARNT). This complex binds to genes with a XRE within the promoter and initiates expression of detoxifying enzymes involved in phase I and II xenobiotic metabolism [40].

A limitation of our study is the chance of exposure misclassification. Information about maternal smoking during pregnancy was based on self-report and is not verifiable. A possibility to overcome this limitation is the determination of the cotinine concentration in plasma or urine of the mother. Nevertheless, previous studies demonstrated that this would not be superior to self-reported smoking habit in pregnant women [2]. We acknowledge the fact that we cannot fully exclude residual or unmeasured confounding by other factors that could be associated with both tobacco smoke exposure and placental molecular signatures. Although a causal relationship exists between prenatal tobacco smoke exposure and low birth weight or preterm birth, not all infants exposed to tobacco smoke develop these adverse perinatal outcomes. It is therefore reasonable to assume that several interactions exists between tobacco smoke exposure and biochemical, genetic, and epigenetic factors which make the fetus more susceptible to changes in fetal programming.

Our findings are of clinical relevance because responses of mitochondrial DNA and changes in the fetal methylome are plausible alterations that may underlie the adverse effect of tobacco smoke exposure on birth weight. They increase our knowledge on the mechanisms of perturbations in the fetoplacental exchange that might lie at basis of low birth weight and, hence, may be used in the broader sense of clinical context.

## Conclusions

This study provides epidemiological evidence of molecular changes in placental tissue that can serve as molecular signatures of exposure to tobacco smoke during pregnancy. Whether the molecular signatures described in our study may be related to early developmental changes in Belgian children will be investigated in the ongoing follow-up study of the ENVIR*ON*AGE birth cohort.

## Additional file

**Additional file 1.** Additional table and figure.

## Abbreviations

*ACTB*: beta actin
Ahr: aryl hydrocarbon receptor
CI: confidence interval
*CYP1A1*: cytochrome P450, family 1, subfamily A, polypeptide 1
*D*-*loop*: displacement loop
ENVIR*ON*AGE: ENVIRonmental influence *ON* early AGEing
*MT*-*ND1*: mitochondrial encoded NADH dehydrogenase 1
*MTF3212/R3319*: mitochondrial forward primer from nucleotide 3212 and reverse primer from nucleotide 3319
*MT*-*RNR1*: mitochondrial region RNR1
mtDNA: mitochondrial DNA
PAH: polycyclic hydrocarbon
qPCR: quantitative real-time polymerase chain reaction
*RPLP0*: acidic ribosomal phosphoprotein P0
