# Supplementary material for: Placental mitochondrial DNA and CYP1A1 gene methylation as molecular signatures for tobacco smoke exposure in pregnant women and the relevance for birth weight
Source: J Transl Med. 2017 Jan 4;15:5. doi: 10.1186/s12967-016-1113-4 (PMC5209876; doi:10.1186/s12967-016-1113-4)
Supplement: Supplementary file 1 — Additional file 1. Additional table and figure. [file 12967_2016_1113_MOESM1_ESM.docx]

**Placental mitochondrial DNA and *CYP1A1* gene methylation as molecular signatures for tobacco smoke exposure in pregnant women and the relevance for birth weight**

Bram G. Janssen, Wilfried Gyselaers, Hyang-Min Byun, Harry A. Roels, Ann Cuypers, Andrea A. Baccarelli, Tim S. Nawrot

Table of Contents

**[Table S1.](#_Toc462152404)** [Bisulfite-Pyrosequencing, mitochondrial and nuclear primer sequence information based upon Assembly GRCh37/hg19 of the UCSC genome browser. 2](#_Toc462152404)

**[Figure S1.](#_Toc462152405)** [Pearson correlation coefficients between CpG sites of](#_Toc462152405) *[CYP1A1](#_Toc462152405)* [gene in placental tissue (](#_Toc462152405)*[n](#_Toc462152405)* [= 382). 4](#_Toc462152405)

| Table S1. Bisulfite-pyrosequencing, mitochondrial and nuclear primer sequence information based upon Assembly GRCh37/hg19 of the UCSC genome browser. | | | | | |
| --- | --- | --- | --- | --- | --- |
| **Gene symbol** | **Chr** | **Amplicon (start-end)** | **Primer set (Forward / Reverse / Sequence)**^†^ | **Amplicon (bp)** | **Target CpGs** |
| **Pyrosequencing** |  |  |  |  |  |
| *CYP1A1* | 15 | 75013061- | F: 5’-TGTTATAGGGTTTTTAGGAAAAA-3’ | 147 | 4 |
|  |  | 75017877 | R: 5’-AAATTATTTTCTAACCTAAACCAAC-3’ |  |  |
|  |  |  | S: 5’-AAAAAAAGTTGTATTTG-3’ |  |  |
| *MT-RNR1* | M | 1191- | F: 5’-TTTTTAGAGGAGTTTGTTTTGTAAT-3’ | 176 | 2 |
|  | (+) | 1366 | R: 5’-ATAACCCATTTCTTACCACCTCATA-3’ |  |  |
|  |  |  | S: 5’-AGTTTGTTTTGTAAT-3’ |  |  |
| *D-loop* | M | 6- | F: 5’-TGTGTAGATATTTAATTGTTATTA-3’ | 254 | 3 |
|  | (-) | 259 | R: 5’-CAAATCTATCACCCTATTAACCAC-3’ |  |  |
|  |  |  | S: 5’-TAATTAATTAATATATTT-3’ |  |  |
| **mtDNA content** |  |  |  |  |  |
| *MTF3212/R3319* | M | 3213- | F:5’-CACCCAAGAACAGGGTTTGT-3’ | 108 | - |
|  |  | 3320 | R:5’-TTAACAACATACCCATGGCCA-3’ |  |  |
| *MT-ND1* | M | 3314- | F:5’-ATGGCCAACCTCCTACTCCT-3’ | 115 | - |
|  |  | 3428 | R:5’-AAAGGCCCCAACGTTGTAG-3’ |  |  |
| *RPLP0* | 12 | 120636904- | F:5’-CCCAATTGTCCCCTTACCT-3’ | 85 | - |
|  |  | 120636988 | R:5’-GAACACAAAGCCCACATTCC-3’ |  |  |
| *ACTB* | 7 | 5567833- | F:5’-ACTCTTCCAGCCTTCCTTCC-3’ | 102 | - |
|  |  | 5567934 | R:5’-TGTGGAAGCTAAGTCCTGCC-3’ |  |  |
| ^†^ The underlined sequence is the biotinylated primer.  Abbreviations: Cytochrome P450, family 1, subfamily A, polypeptide 1 (*CYP1A1*); Mitochondrial region RNR1 (*MT-RNR1*), Displacement loop (*D-loop*); mitochondrial forward primer from nucleotide 3212 and reverse primer from nucleotide 3319 (*MTF3212/R3319*); Mitochondrial encoded NADH dehydrogenase 1 (*MT-ND1*); Acidic ribosomal phosphoprotein P0 (*RPLP0*); Beta actin (*ACTB*). | | | | | |

|  | Placental tissue | | | | |  |
| --- | --- | --- | --- | --- | --- | --- |
|  | 1 | 1 |  |  |  |  |
|  | 2 | 0.93 | 1 |  |  |  |
|  | 3 | 0.71 | 0.81 | 1 |  |  |
|  | 4 | 0.75 | 0.85 | 0.83 | 1 |  |
|  | CpGs | 1 | 2 | 3 | 4 |  |

**Figure S1.** Pearson correlation coefficients between CpG sites of the *CYP1A1* gene in placental tissue (*n* = 382). All *p*-values were < 0.0001.
